# Comparative clinical efficacy and immunological modulation of subcutaneous vs. sublingual allergen immunotherapy in children with house dust mite-induced allergic asthma: a retrospective cohort study

**DOI:** 10.3389/fmed.2026.1909274

**Published:** 2026-09-23

**Authors:** Chunyun Zeng, Huajie Yan, Huiting Xu, Yunan Yang, Wei Dong

**Affiliations:** Department of Pediatrics, Nanxiang Branch of Ruijin Hospital, Shanghai, China

**Keywords:** allergen immunotherapy, allergic asthma, house dust mite, subcutaneous immunotherapy, sublingual immunotherapy

## Abstract

**Objectives:**

The current study compared the clinical efficacy and immunological effects of subcutaneous immunotherapy (SCIT) and sublingual immunotherapy (SLIT) in children with house dust mite (HDM)-induced allergic asthma and explored immune markers associated with treatment response.

**Methods:**

The study retrospectively included 125 children with HDM-induced allergic asthma, of which 53 patients received SCIT and 72 patients received SLIT. Clinical outcomes, pulmonary function, and immunological parameters were evaluated at baseline and after 12 months of treatment. Immunological assessments included total IgE, HDM-specific IgE, IgG4, cytokines, peripheral eosinophils, and fractional exhaled nitric oxide (FeNO). Multivariable regression and correlation analyses were performed to identify factors associated with clinical improvement.

**Results:**

Both SCIT and SLIT significantly improved asthma control and pulmonary function. ACT scores increased, whereas FeNO levels and eosinophil counts decreased in both groups. IgG4 and IL-10 levels increased significantly, while IL-4 and IL-5 decreased. No significant differences were observed between SCIT and SLIT in the magnitude of these improvements. In exploratory regression analyses, changes in IgG4, IL-10, and IL-4 were associated with improvement in asthma control.

**Conclusions:**

In this retrospective cohort, SCIT and SLIT were associated with similar improvements in asthma control, selected pulmonary-function measures, and immune markers over the 12-month observation period. Changes in concomitant asthma medications during follow-up could not be accounted for and may have influenced the observed outcomes. The study did not assess the long-term sustainability of these benefits or post-treatment effects; therefore, longer prospective follow-up is required to evaluate the comparative long-term efficacy of the two treatment modalities.

## Introduction

Allergic asthma is one of the most common chronic respiratory diseases in children and continues to impose a substantial health burden worldwide. Over the past few decades, epidemiological surveys document a noticeable rise in asthma prevalence among children and adolescents, particularly in rapidly urbanizing regions (1). Asthma in childhood is strongly associated with allergic sensitization, and inhalant allergens encountered during early life are considered key contributors to the initiation and persistence of airway inflammation (2). Among the various indoor allergens, house dust mite (HDM) has been identified as one of the most important triggers of allergic respiratory diseases, the allergens of which are widely distributed in indoor environments such as bedding, carpets, and furniture, making exposure difficult to avoid even with environmental control measures (3).

HDM sensitization is frequently detected in pediatric patients with asthma and is strongly associated with both disease severity and persistence. The major mite species involved in allergic diseases are *Dermatophagoides pteronyssinus* and *Dermatophagoides farinae*, which produce potent allergenic proteins capable of activating both innate and adaptive immune pathways (4). In sensitized individuals, repeated allergen exposure leads to IgE-mediated immune responses characterized by activation of mast cells and basophils, increased production of type-2 cytokines, and recruitment of eosinophils into the airway mucosa. These processes contribute to airway inflammation, bronchial hyperresponsiveness, and reversible airflow limitation, which are hallmarks of allergic asthma (5). Longitudinal studies have also demonstrated that early-life allergen exposure and sensitization may influence the natural course of asthma and increase the likelihood of persistent disease into adolescence and adulthood (6).

The current management of allergic asthma relies primarily on pharmacological treatments that aim to control symptoms and prevent exacerbations. Inhaled corticosteroids (ICS) remain the cornerstone therapy and have been shown to significantly improve lung function and reduce airway inflammation in most patients (7). However, conventional pharmacological treatment does not address the underlying allergic mechanisms responsible for disease development. Consequently, many patients require long-term medication to maintain symptom control, and the disease may relapse once treatment is discontinued. For this reason, therapeutic strategies capable of modifying the natural history of allergic disease have attracted increasing attention.

Allergen immunotherapy (AIT) is the only treatment that can induce long-term immune tolerance to specific allergens. It involves the administration of gradually increasing doses of allergen extracts to modify immune responses and reduce allergic inflammation (8). The immunological effects of AIT involve multiple mechanisms, including suppression of Th2-driven immune responses, induction of regulatory T cells, and increased production of allergen-specific IgG4 antibodies that can block IgE-mediated allergic reactions (9). Over time, these immunological changes lead to reduced allergen sensitivity, improved clinical symptoms, and decreased medication use. Evidence from systematic reviews and meta-analyses has confirmed that allergen immunotherapy can provide significant benefits for patients with allergic asthma and allergic rhinitis (10). Two major forms of allergen immunotherapy are widely used in clinical practice: subcutaneous immunotherapy (SCIT) and sublingual immunotherapy (SLIT). SCIT involves periodic injections of allergen extracts and has been used for more than a century. Numerous clinical trials have demonstrated that SCIT can significantly reduce asthma symptoms and medication requirements in allergic patients (11). In addition, long-term follow-up studies suggest that the clinical benefits of immunotherapy may persist even after treatment discontinuation, indicating a potential disease-modifying effect (12). Nevertheless, SCIT requires regular clinic visits and carries a small risk of systemic allergic reactions. Thus, SLIT was introduced as an alternative method designed to improve treatment safety and convenience. In SLIT, allergen extracts are administered under the tongue and can usually be taken at home after the first supervised dose. Compared with SCIT, SLIT is generally associated with fewer systemic adverse reactions and better patient adherence, particularly among children (13). Several randomized controlled trials and meta-analyses have shown that SLIT can effectively reduce symptoms of allergic rhinitis and asthma, although some studies have reported slightly lower efficacy compared with SCIT (14). In recent years, HDM-specific SLIT tablets have also demonstrated clinical efficacy in patients with HDM-driven allergic asthma in large randomized trials (15).

Despite the growing body of evidence supporting allergen immunotherapy, several questions remain unresolved. In particular, direct comparisons between SCIT and SLIT in pediatric populations are relatively limited, and the immunological changes associated with these therapies in real-world clinical settings remain incompletely understood. Furthermore, not all patients respond equally to immunotherapy, and identifying biomarkers that can predict treatment response has become an important area of research (16, 17). Biomarkers such as allergen-specific IgE, IgG4, and eosinophil counts have been proposed as potential indicators of treatment efficacy, although their predictive value remains under investigation (18). Therefore, the present retrospective cohort study aimed to compare the clinical efficacy and immunological effects of SCIT and SLIT in children with HDM-induced allergic asthma. By analyzing changes in asthma control, lung function, and immunological markers during a 12-month treatment period, this study also sought to explore immunological factors associated with treatment response. The findings may contribute to a better understanding of immunological changes during allergen immunotherapy in pediatric allergic asthma.

## Methods

### Study design and population

This study was designed as a retrospective observational cohort study to evaluate the clinical efficacy and immunological effects of HDM allergen immunotherapy in children with allergic asthma. Patients who initiated immunotherapy between June 2023 and December 2024 were retrospectively enrolled, and 12-month follow-up data were collected through December 2025. A total of 125 pediatric patients diagnosed with HDM-induced allergic asthma were enrolled from hospital records. All participants had confirmed sensitization to HDM allergens and were receiving allergen immunotherapy as part of routine clinical management. The enrolled 125 children were identified from the available hospital records during the study period, including 53 children in the SCIT group and 72 children in the SLIT group. Given the retrospective design, the analysis did not include a prespecified sample-size calculation.

This retrospective observational cohort study was performed based on hospital records from children who received HDM allergen immunotherapy as part of routine clinical management. No treatment was allocated by the investigators for this retrospective analysis. Patients were classified according to the immunotherapy modality documented in their medical records: 53 children received SCIT and 72 received SLIT. Treatment allocation was not randomized. The available retrospective records enabled classification of patients according to the treatment received but did not provide a standardized treatment-allocation algorithm. Baseline demographic and clinical characteristics, including age, sex, disease duration, family history of allergy, allergic rhinitis, asthma control, pulmonary-function measures, and immunological parameters, were collected for both groups. Patients were followed for 12 months after initiation of immunotherapy, and clinical and laboratory assessments were performed at baseline and during follow-up. This study was conducted in accordance with the ethical standards of the Declaration of Helsinki. Ethical approval was obtained from the Ethics Committee of Nanxiang Hospital.

### Inclusion and exclusion criteria

Participants were eligible for inclusion if they met the following criteria: age between 5 and 14 years; diagnosis of allergic asthma according to Global Initiative for Asthma (GINA) criteria; sensitization to house dust mite allergens confirmed by serum specific IgE or skin prick test; stable asthma condition without acute exacerbation within the previous four weeks; availability of evaluable clinical data at both baseline and the 12-month follow-up assessment. Patients were excluded if they met any of the following conditions: Severe uncontrolled asthma; Presence of other chronic respiratory diseases such as bronchiectasis or cystic fibrosis; Severe cardiovascular, autoimmune, or systemic diseases; Previous allergen immunotherapy within the past three years; Missing baseline or 12-month follow-up data for the outcomes assessed in this study. Finally, the analytical cohort comprised 125 children with evaluable baseline and 12-month data. All included participants had complete data for the variables analyzed at the 12-month assessment. Patients with missing baseline or follow-up data were not included in the analytical cohort.

### Immunotherapy protocol

#### SCIT treatment

Children in the SCIT group received Novo-Helisen® Depot, a standardized HDM allergen extract containing Dermatophagoides farinae and Dermatophagoides pteronyssinus (Allergopharma GmbH & Co. KG, Reinbek, Germany). The treatment included a weekly dose-escalation phase comprising 14 weekly injections using three concentrations of the extract (50, 500, and 5,000 TE/mL), followed by maintenance treatment with 1.0 mL of the 5,000 TE/mL preparation every 4 weeks. All injections were administered in the hospital under medical supervision, and patients were observed for at least 30 min after each injection.

#### SLIT treatment

Patients in the SLIT group received standardized Dermatophagoides farinae sublingual drops (Chanllergen®, Zhejiang Wolwo Bio-Pharmaceutical Co., Ltd., Zhejiang, China). The product consisted of five sequential concentrations of allergen extract: 1, 10, 100, 333, and 1,000 μg/mL. Treatment began with a 3-week dose-escalation phase using the first three concentrations, followed by once-daily maintenance treatment. Children aged <14 years received 3 drops of the 333 μg/mL preparation daily, whereas patients aged ≥14 years received 2 drops of the 1,000 μg/mL preparation daily. The first dose was administered under physician supervision, and treatment was subsequently continued at home. The drops were retained under the tongue for approximately 1–3 min before swallowing. Tablets were not used in this study.

#### Treatment adherence and follow-up

Adherence to SCIT was assessed from hospital injection records. For SLIT, caregivers were instructed to supervise daily administration and record missed doses; this information was reviewed during follow-up visits. However, adherence was not quantified using a validated measure and was not included as an independent variable in the comparative analyses.

### Clinical evaluation

#### Asthma control test (ACT)

Asthma control was assessed using the Asthma Control Test (ACT). The ACT questionnaire consists of five items evaluating symptom frequency, activity limitation, and rescue-medication use during the previous four weeks. Scores range from 5 to 25, with higher scores reflecting better asthma control. Rescue-medication use was considered only as one component of the composite ACT score. No separate longitudinal medication-use score or analysis of controller-medication requirements was performed.

### Pulmonary function testing

Pulmonary function was assessed using a computerized spirometry system (e.g., Jaeger MasterScreen PFT System, CareFusion, Germany) according to the recommendations of the American Thoracic Society and the European Respiratory Society. All tests were performed by trained technicians in a standardized pulmonary function laboratory. Before testing, children were instructed to avoid short-acting bronchodilators for at least 6 h and long-acting bronchodilators for at least 24 h whenever possible. After a brief demonstration, each child performed a series of forced expiratory maneuvers while wearing a nose clip and using a disposable mouthpiece. During each maneuver, the participant was instructed to inhale fully to total lung capacity and then exhale forcefully and continuously until no further air could be expelled. Each subject performed at least three acceptable maneuvers, and the best value meeting quality criteria was used for analysis. **T**he following spirometric parameters were recorded.

#### Forced expiratory volume in one second (FEV1)

FEV1 represents the volume of air exhaled during the first second of forced expiration and reflects airway obstruction. The measured value was expressed both as an absolute value (L) and as a percentage of the predicted value (FEV1%).

#### Forced vital capacity (FVC)

FVC represents the total volume of air that can be forcibly exhaled after maximal inhalation. This parameter was recorded in liters and compared with predicted values adjusted for age, sex, height, and ethnicity.

#### FEV1/FVC ratio

The ratio of FEV1 to FVC was calculated automatically by the spirometry software and used as an indicator of airflow limitation.

#### Peak expiratory flow (PEF)

PEF represents the highest expiratory flow achieved during forced exhalation and reflects large airway function.

Pulmonary function measurements were performed at baseline before initiation of immunotherapy and repeated at the 12-month follow-up visit. All results were reviewed to ensure compliance with quality control standards.

### Immunological measurements

Peripheral venous blood samples were collected from each participant at baseline and after 12 months of treatment. Blood samples were drawn in the morning after an overnight fast to minimize potential biological variation. Serum was separated by centrifugation at 3,000 rpm for 10 min and stored at −80 °C until analysis.

#### Serum total IgE

Serum total immunoglobulin E (IgE) levels were measured using a chemiluminescent immunoassay system (e.g., ImmunoCAP, Thermo Fisher Scientific, Sweden). The assay was performed according to the manufacturer's protocol. Briefly, serum samples were incubated with anti-IgE antibodies immobilized on a solid phase, followed by enzyme-labeled detection antibodies. Chemiluminescent signals generated during the reaction were measured using an automated analyzer, and IgE concentrations were calculated using calibration curves. Results were expressed in international units per milliliter (IU/mL).

#### House dust mite–specific IgE

Specific IgE antibodies against HDM allergens (*Dermatophagoides pteronyssinus* and *Dermatophagoides farinae*) were quantified using a fluorescence enzyme immunoassay system (ImmunoCAP). Serum samples were incubated with allergen-coated solid phases to capture allergen-specific IgE antibodies. After washing, enzyme-labeled anti-IgE antibodies were added, and fluorescence intensity was measured to determine antibody concentrations. Results were expressed in kilo units of allergen-specific IgE per liter (kUA/L) and categorized into standard sensitization grades according to established guidelines.

#### Allergen-specific IgG4

Serum HDM-specific IgG4 levels were measured using an enzyme-linked immunosorbent assay (ELISA). Microtiter plates coated with HDM allergen extracts were incubated with diluted serum samples. After washing, horseradish peroxidase–conjugated anti-human IgG4 antibodies were added as detection antibodies. The reaction was developed using tetramethylbenzidine substrate, and optical density was measured at 450 nm using a microplate reader. IgG4 concentrations were calculated based on standard curves generated using reference standards. Increased IgG4 levels are considered indicative of immunological tolerance induced by allergen immunotherapy.

#### Peripheral blood eosinophil count

Peripheral blood eosinophil counts were determined using an automated hematology analyzer (e.g., Sysmex XN-1000). Whole blood samples collected in EDTA anticoagulant tubes were analyzed within two hours after collection. The analyzer provided both the absolute eosinophil count (×10^9^/L) and the percentage of eosinophils among total leukocytes. Elevated eosinophil levels were considered indicative of allergic inflammation.

#### Cytokine measurement

Serum cytokine concentrations were measured using commercially available enzyme-linked immunosorbent assay kits (ELISA) following the manufacturer's instructions. The cytokines analyzed in this study included interleukin-4 (IL-4), interleukin-5 (IL-5), interleukin-10 (IL-10), and interferon-γ (IFN-γ). Briefly, serum samples and standard solutions were added to wells coated with specific capture antibodies. After incubation and washing, biotinylated detection antibodies were added, followed by streptavidin-conjugated enzyme. Color development was achieved using a chromogenic substrate solution, and absorbance was measured at 450 nm using a microplate reader. Cytokine concentrations were calculated based on standard curves generated from known concentrations. IL-4 and IL-5 were selected as markers of Th2-mediated allergic inflammation, while IFN-γ represented Th1 immune responses. IL-10 was analyzed as an indicator of regulatory immune activity associated with immune tolerance during allergen immunotherapy.

### Fractional exhaled nitric oxide (FeNO)

Fractional exhaled nitric oxide (FeNO) was measured as a non-invasive biomarker of airway inflammation. FeNO levels were assessed using a portable nitric oxide analyzer (e.g., NIOX VERO, Circassia Pharmaceuticals, UK) according to the recommendations of the American Thoracic Society. During the test, children were instructed to inhale to total lung capacity through a mouthpiece and then exhale slowly at a constant flow rate of 50 mL/s for approximately 10 s. The analyzer continuously measured nitric oxide concentration in exhaled air. Each subject performed two acceptable measurements, and the mean value was recorded for analysis. FeNO values were expressed in parts per billion (ppb). Increased FeNO levels were considered indicative of eosinophilic airway inflammation. Because detailed longitudinal information on inhaled or systemic corticosteroid use was unavailable, the FeNO analyses were not adjusted for corticosteroid exposure.

### Safety monitoring

Adverse reactions occurring during immunotherapy were monitored and recorded throughout the study period. For SCIT, patients were observed in the clinic for at least 30 min after each injection to detect possible immediate hypersensitivity reactions. For SLIT, patients and their guardians were instructed to report any local or systemic reactions occurring during treatment. Adverse events were classified as local reactions (such as redness, swelling, or itching at the injection site) or systemic reactions (including urticaria, wheezing, angioedema, or anaphylaxis). The severity of reactions was graded according to established immunotherapy safety guidelines.

### Statistical analysis

All statistical analyses were performed using SPSS software (version 26.0). Continuous variables were expressed as mean ± standard deviation (SD), whereas categorical variables were presented as numbers and percentages. Baseline characteristics were compared between groups using independent-sample t-tests for continuous variables and chi-square tests for categorical variables. Within-group changes from baseline to 12 months were assessed using paired t-tests. For formal between-group comparisons at 12 months, analysis of covariance was performed for selected outcomes: ACT score, FEV1, FVC, serum IgG4, IL-4, IL-10, FeNO, and peripheral blood eosinophil count. These outcomes were selected because they represent asthma control, core lung-function measures, and immunological or inflammatory markers relevant to the proposed effects of allergen immunotherapy. FEV1/FVC ratio, PEF, total IgE, HDM-specific IgE, IL-5, and IFN-*γ* were not included in the formal ANCOVA analyses. The 12-month value was specified as the dependent variable, treatment group was specified as the independent variable, and the corresponding baseline value was included as a covariate. For the logistic regression analysis, treatment response was defined as an ACT score ≥20 at 12 months, which indicates well-controlled asthma. Candidate variables in the exploratory logistic regression model were selected based on clinical relevance and biological plausibility. Because the number of treatment-response events was limited relative to the number of candidate variables, this analysis was intended to explore associations and was not developed or validated as a clinical prediction model. As a sensitivity analysis, clinically meaningful improvement was defined as an increase in ACT score of ≥3 points from baseline. Multivariable regression analyses were exploratory association analyses and were not intended for prediction-model development or validation. Adjusted mean differences between SCIT and SLIT, 95% confidence intervals, and two-sided *P* values were reported. To control the false-discovery rate arising from multiple comparisons within each analysis, *P* values were adjusted using the Benjamini–Hochberg procedure. Adjusted *P* values are reported in the tables and were used for interpretation. A two-sided adjusted *P* value <0.05 was considered statistically significant. Because the study was retrospective and was not designed as an equivalence or non-inferiority trial, non-significant between-group comparisons were not interpreted as evidence of equivalent efficacy. Analyses were conducted using complete cases. No data imputation was performed because no missing values were present for the variables included in the analytical dataset.

## Results

### Baseline characteristics of the study population

A total of 125 children with house dust mite–induced allergic asthma were included in the analysis. Among them, 53 patients received SCIT treatment and 72 patients received SLIT treatment. As summarized in Table 1, there were no significant differences between the two groups in baseline demographic characteristics, clinical severity, pulmonary function, or immunological parameters. The mean age of the participants was 8.47 ± 2.20 years in the SCIT group and 9.14 ± 2.17 years in the SLIT group (*P* = 0.092). The proportion of male patients was comparable between the two groups (54.7% vs. 51.4%, *P* = 0.852). Similarly, disease duration and the prevalence of allergic rhinitis did not differ significantly between groups (both *P* > 0.05). Baseline asthma control, assessed by the ACT score, was also similar between the SCIT and SLIT groups (16.37 ± 2.27 vs. 15.80 ± 2.56, *P* = 0.201). No significant differences were observed in baseline pulmonary function parameters, including FEV1, FVC, FEV1/FVC ratio, and PEF (all *P* > 0.05). Furthermore, immunological indicators such as total IgE, HDM-specific IgE, IgG4 levels, cytokine profiles (IL-4, IL-5, IL-10, IFN-*γ*), FeNO, and peripheral blood eosinophil counts were comparable between groups. However, because treatment allocation was not randomized, similarity in the measured baseline variables does not exclude selection bias or residual confounding from unmeasured factors.

**Table 1:** Baseline characteristics of the study population.

Variable	SCIT (*n* = 53)	SLIT (*n* = 72)	BH-adjusted *P* value
Age (years)	8.47 ± 2.20	9.14 ± 2.17	0.092
Male, *n* (%)	29 (54.7%)	37 (51.4%)	0.852
Disease duration (years)	3.57 ± 1.89	3.51 ± 1.57	0.86
Family history of allergy, *n* (%)	32 (60.4%)	40 (55.6%)	0.722
Allergic rhinitis, *n* (%)	29 (54.7%)	46 (63.9%)	0.395
ACT score	16.37 ± 2.27	15.80 ± 2.56	0.201
FEV1 (% predicted)	80.95 ± 6.57	81.48 ± 6.04	0.641
FVC (% predicted)	90.37 ± 4.81	91.04 ± 5.77	0.888
FEV1/FVC	0.75 ± 0.05	0.75 ± 0.05	0.63
PEF (% predicted)	81.99 ± 7.09	82.49 ± 5.67	0.659
Total IgE (IU/mL)	295.72 ± 129.49	283.90 ± 127.36	0.612
HDM-specific IgE (kUA/L)	16.92 ± 6.79	17.58 ± 5.57	0.551
IgG4 (mg/L)	0.41 ± 0.18	0.40 ± 0.17	0.838
IL-4 (pg/mL)	8.51 ± 1.92	8.33 ± 1.99	0.613
IL-5 (pg/mL)	6.41 ± 1.59	6.47 ± 1.69	0.859
IL-10 (pg/mL)	2.95 ± 1.28	2.85 ± 1.26	0.641
IFN-*γ* (pg/mL)	5.67 ± 1.83	5.91 ± 1.50	0.433
FeNO (ppb)	38.34 ± 8.43	38.79 ± 8.14	0.761
Blood eosinophils (×10^9^/L)	0.55 ± 0.17	0.56 ± 0.18	0.812

SCIT, subcutaneous immunotherapy; SLIT, sublingual immunotherapy; ACT, asthma control test; FEV1, forced expiratory volume in one second; FVC, forced vital capacity; PEF, peak expiratory flow; HDM, house dust mite; *P* values were adjusted for multiple comparisons using the Benjamini-Hochberg false-discovery-rate procedure. A two-sided adjusted *P* value <0.05 was considered statistically significant.

### Within-group changes and baseline-adjusted between-group comparisons

Table 2 summarizes within-group changes from baseline to 12 months and baseline-adjusted between-group comparisons for the selected outcomes included in the ANCOVA analyses. Both groups showed significant within-group improvements in ACT score, FEV1, FVC, IgG4, IL-4, IL-10, FeNO, and blood eosinophil count (all *P* < 0.001). In the baseline-adjusted analyses of covariance, no statistically significant between-group differences were observed for any of these eight outcomes.

**Table 2:** Changes from baseline to 12 months and baseline-adjusted between-group comparisons for selected clinical, lung-function, immunological, and inflammatory outcomes.

Outcome	SCIT baseline	SCIT 12 months	BH-adjusted *P* value, within SCIT	SLIT baseline	SLIT 12 months	BH-adjusted *P* value, within SLIT	Adjusted mean difference, SCIT−SLIT	95% CI	BH-adjusted *P* value
ACT score	16.37 ± 2.27	20.92 ± 2.79	<0.001	15.80 ± 2.56	20.51 ± 2.63	<0.001	−0.12	−0.64 to 0.40	0.766
FEV1 (% predicted)	80.95 ± 6.57	89.40 ± 7.28	<0.001	81.48 ± 6.04	90.17 ± 6.97	<0.001	−0.23	−1.30 to 0.84	0.725
FVC (% predicted)	90.37 ± 4.81	96.66 ± 4.37	<0.001	91.04 ± 5.77	97.04 ± 6.48	<0.001	0.25	−0.67 to 1.18	0.587
IgG4 (mg/L)	0.41 ± 0.18	1.05 ± 0.30	<0.001	0.40 ± 0.17	1.02 ± 0.31	<0.001	0.02	−0.07 to 0.12	0.595
IL-4 (pg/mL)	8.51 ± 1.92	4.82 ± 2.36	<0.001	8.33 ± 1.99	5.12 ± 2.23	<0.001	−0.48	−0.92 to −0.04	0.248
IL-10 (pg/mL)	2.95 ± 1.28	6.31 ± 1.85	<0.001	2.85 ± 1.26	6.33 ± 2.03	<0.001	−0.13	−0.64 to 0.39	0.624
FeNO (ppb)	38.34 ± 8.43	25.88 ± 8.93	<0.001	38.79 ± 8.14	26.63 ± 8.15	<0.001	−0.35	−1.94 to 1.25	0.669
Blood eosinophils (×10⁹/L)	0.55 ± 0.17	0.32 ± 0.19	<0.001	0.56 ± 0.18	0.33 ± 0.21	<0.001	0	−0.03 to 0.03	0.890

Formal baseline-adjusted ANCOVA was performed only for the eight outcomes presented in this table: ACT score, FEV1, FVC, IgG4, IL-4, IL-10, FeNO, and blood eosinophil count. FEV1/FVC ratio, PEF, total IgE, HDM-specific IgE, IL-5, and IFN-γ were not included in the formal ANCOVA analyses.

SCIT, subcutaneous immunotherapy; SLIT, sublingual immunotherapy; ACT, asthma control test; FEV1, forced expiratory volume in one second; FVC, forced vital capacity.

Between-group comparisons were performed using analysis of covariance adjusted for baseline values. *P* values were adjusted for multiple comparisons using the Benjamini–Hochberg false-discovery-rate procedure. A two-sided adjusted *P* value <0.05 was considered statistically significant.

The mean ACT score increased by 4.55 points in the SCIT group and 4.71 points in the SLIT group, exceeding the commonly used 3-point minimal clinically important difference for the ACT. In the sensitivity analysis, clinically meaningful improvement, defined as an ACT increase of ≥3 points from baseline, was observed in 44 of 53 children (83.0%) in the SCIT group and 62 of 72 children (86.1%) in the SLIT group, with no significant between-group difference (*P* = 0.634). Mean FEV1 increased by 8.45 percentage points of predicted value in the SCIT group and 8.69 percentage points in the SLIT group.

### Factors associated with clinical improvement

In the exploratory multivariable linear regression analysis, changes in IgG4, IL-4, and IL-10 were statistically associated with changes in ACT score over the 12-month follow-up period (Table 3). These associations do not establish causal mechanisms. After adjustment for the covariates included in the model, *Δ*IgG4 was positively associated with *Δ*ACT (*β* = 0.92, *P* = 0.002), and *Δ*IL-10 was also positively associated with *Δ*ACT (*β* = 0.56, *P* < 0.001). In contrast, *Δ*IL-4 was negatively associated with *Δ*ACT (*β* = −0.41, *P* = 0.008). Age, disease duration, baseline FEV1, total IgE, and treatment group were not independently associated with *Δ*ACT. In this exploratory model, these immunological changes were statistically associated with improved asthma control during the 12-month observation period.

**Table 3:** Exploratory multivariable linear regression of factors associated with changes in ACT score from baseline to 12 months.

Variable	β	95% CI	BH-adjusted *P* value
Age	0.08	−0.03–0.19	0.16
Disease duration	−0.11	−0.27–0.04	0.15
Baseline FEV1	0.05	−0.02–0.12	0.18
Total IgE	−0.001	−0.002–0.000	0.08
HDM-specific IgE	−0.06	−0.18–0.05	0.28
*Δ*IgG4	0.92	0.35–1.49	0.002
*Δ*IL-4	−0.41	−0.70 to −0.11	0.008
*Δ*IL-10	0.56	0.24–0.88	<0.001
*Δ*FeNO	−0.03	−0.07–0.01	0.11
*Δ*Eosinophils	−0.28	−0.63–0.07	0.12
Treatment group	0.19	−0.44–0.81	0.55

FEV1, forced expiratory volume in one second.

*P* values were adjusted for multiple comparisons using the Benjamini–Hochberg false-discovery-rate procedure. A two-sided adjusted *P* value <0.05 was considered statistically significant. *Δ* indicates the value at 12 months minus the baseline value. The regression coefficients represent the association between each predictor and the change in ACT score during follow-up. Results should be interpreted as exploratory associations.

### Correlation between immunological changes and clinical outcomes

To further examine the relationship between immunological changes and symptom improvement, correlation analyses were performed between changes in immune markers and *Δ*ACT scores (Table 4 and Figure 1). *Δ*IgG4 showed the strongest positive correlation with *Δ*ACT (*r* = 0.48, *P* < 0.001). *Δ*IL-10 was also positively correlated with *Δ*ACT (*r* = 0.41, *P* < 0.001), whereas *Δ*IL-4 was negatively correlated with *Δ*ACT (*r* = −0.37, *P* < 0.001). Changes in FeNO and peripheral blood eosinophil counts were also negatively correlated with *Δ*ACT. These analyses describe associations between immunological changes and asthma-control improvement and do not establish causal or mediating effects.

**Table 4:** Correlation between immunological changes and clinical improvement.

Variable	*r*	BH-adjusted *P* value
*Δ*IgG4	0.48	<0.001
*Δ*IL-10	0.41	<0.001
*Δ*IL-4	−0.37	<0.001
*Δ*IL-5	−0.29	0.002
*Δ*IFN-γ	0.21	0.018
*Δ*FeNO	−0.35	<0.001
*Δ*Eosinophils	−0.33	<0.001

*P* values were adjusted for multiple comparisons using the Benjamini–Hochberg false-discovery-rate procedure. A two-sided adjusted *P* value <0.05 was considered statistically significant.

**Figure 1:**
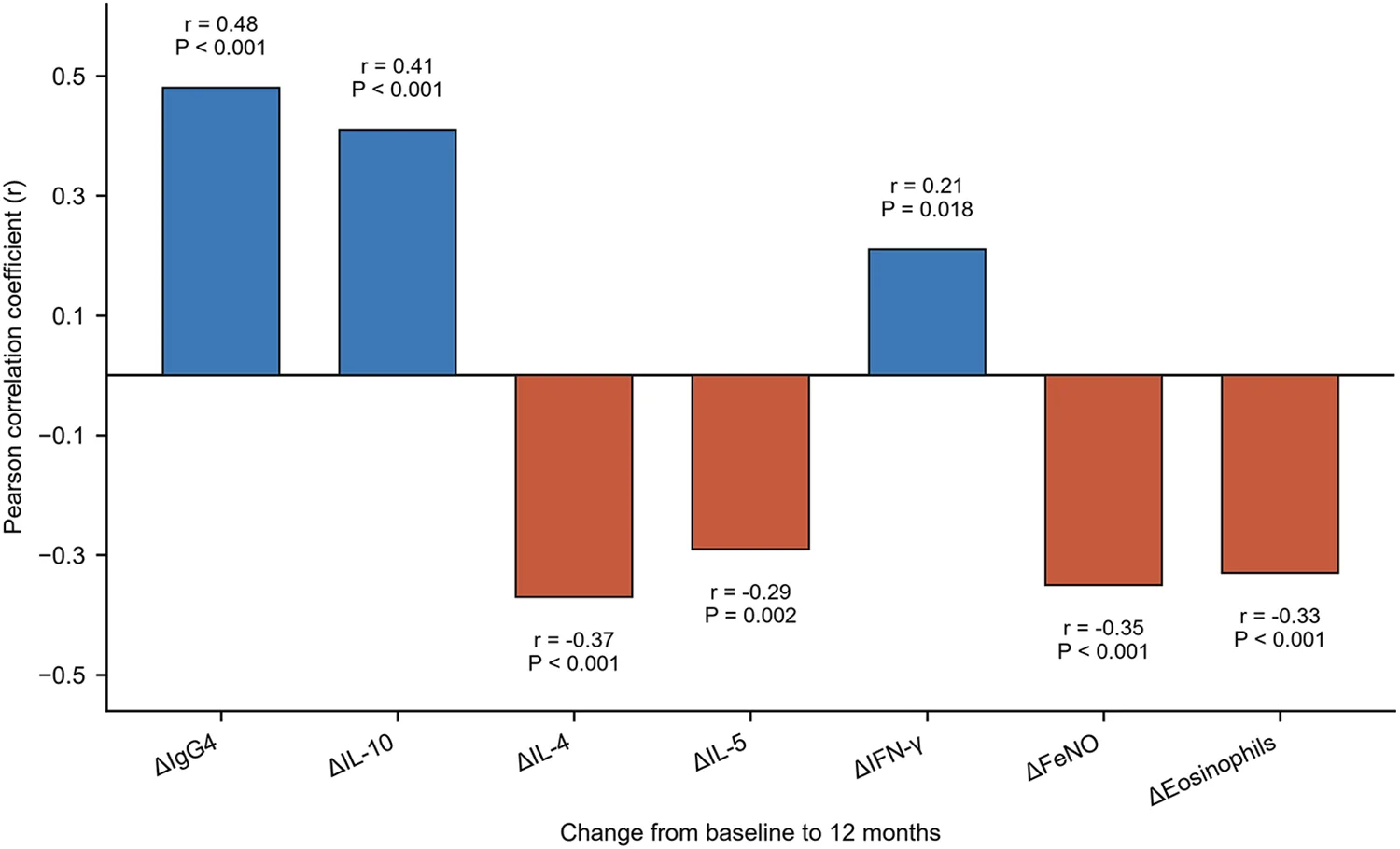
Correlations between 12-month changes in immunological and inflammatory markers and improvement in asthma control. Pearson correlation coefficients (r) show associations with changes in Asthma Control Test score (*Δ*ACT) among all participants (*n* = 125). Positive and negative coefficients indicate greater ACT improvement with increasing and decreasing marker levels, respectively. *Δ*, change from baseline to 12 months; ACT, asthma control test; IgG4, immunoglobulin G4; IL, interleukin; IFN-γ, interferon-gamma; FeNO, fractional exhaled nitric oxide.

The distribution of IgG4 responses following immunotherapy is illustrated in Figure 2, which shows substantial increases in IgG4 levels in both treatment groups. In addition, Figure 3 presents the estimated associations of selected immunological markers with ACT improvement in the exploratory multivariable linear regression model.

**Figure 2:**
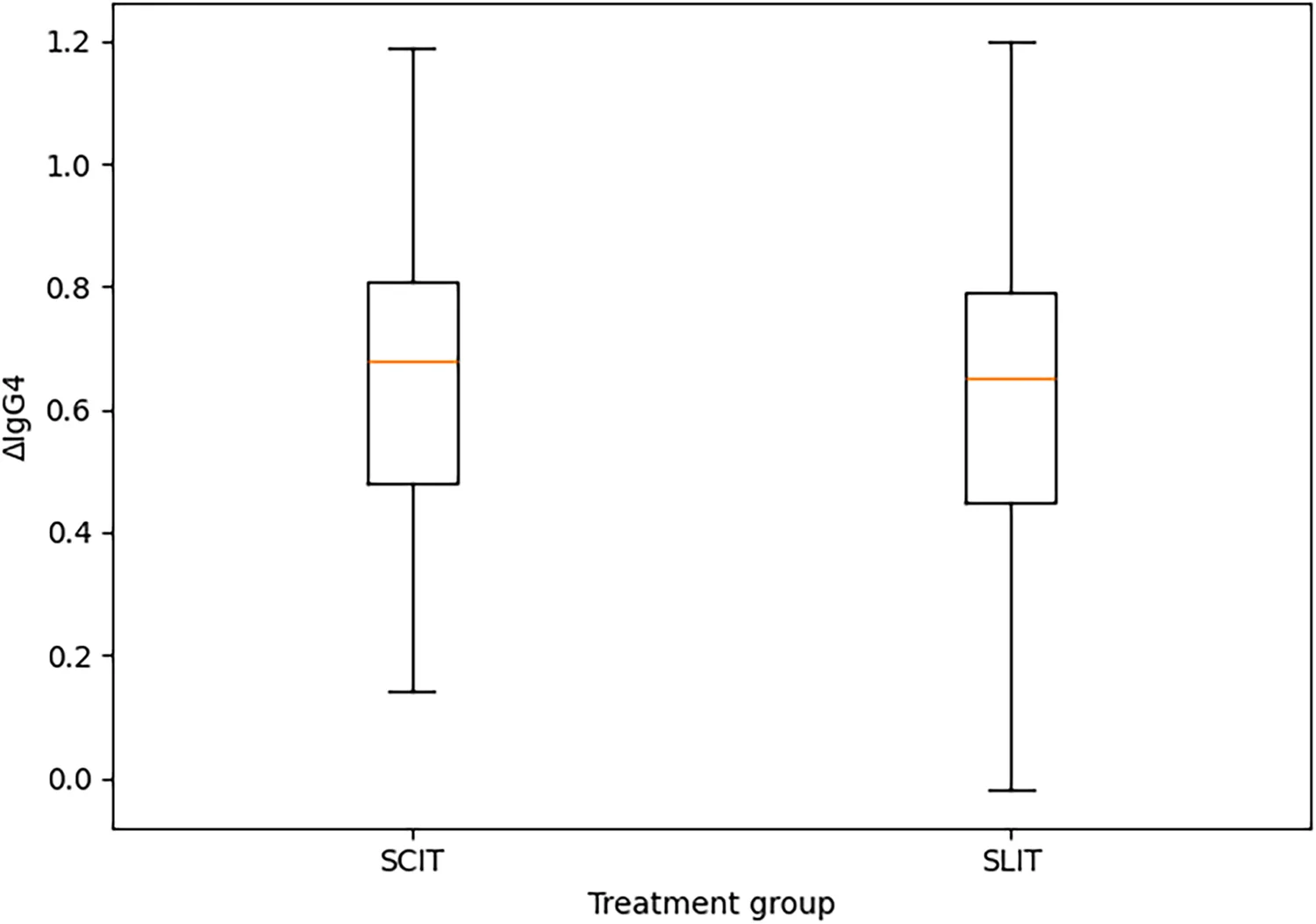
Changes in house dust mite-specific IgG4 after 12 months of immunotherapy. Box plots show the change in serum IgG4 concentration from baseline to 12 months in the SCIT (*n* = 53) and SLIT (*n* = 72) groups. The central line represents the median, the box represents the interquartile range, and whiskers represent the minimum and maximum values. The baseline-adjusted between-group comparison was not statistically significant (adjusted mean difference, 0.02 mg/L; 95% CI, −0.07 to 0.12; BH-adjusted *P* = 0.595). SCIT, subcutaneous immunotherapy; SLIT, sublingual immunotherapy; *Δ*IgG4, change in IgG4 concentration from baseline to 12 months.

**Figure 3:**
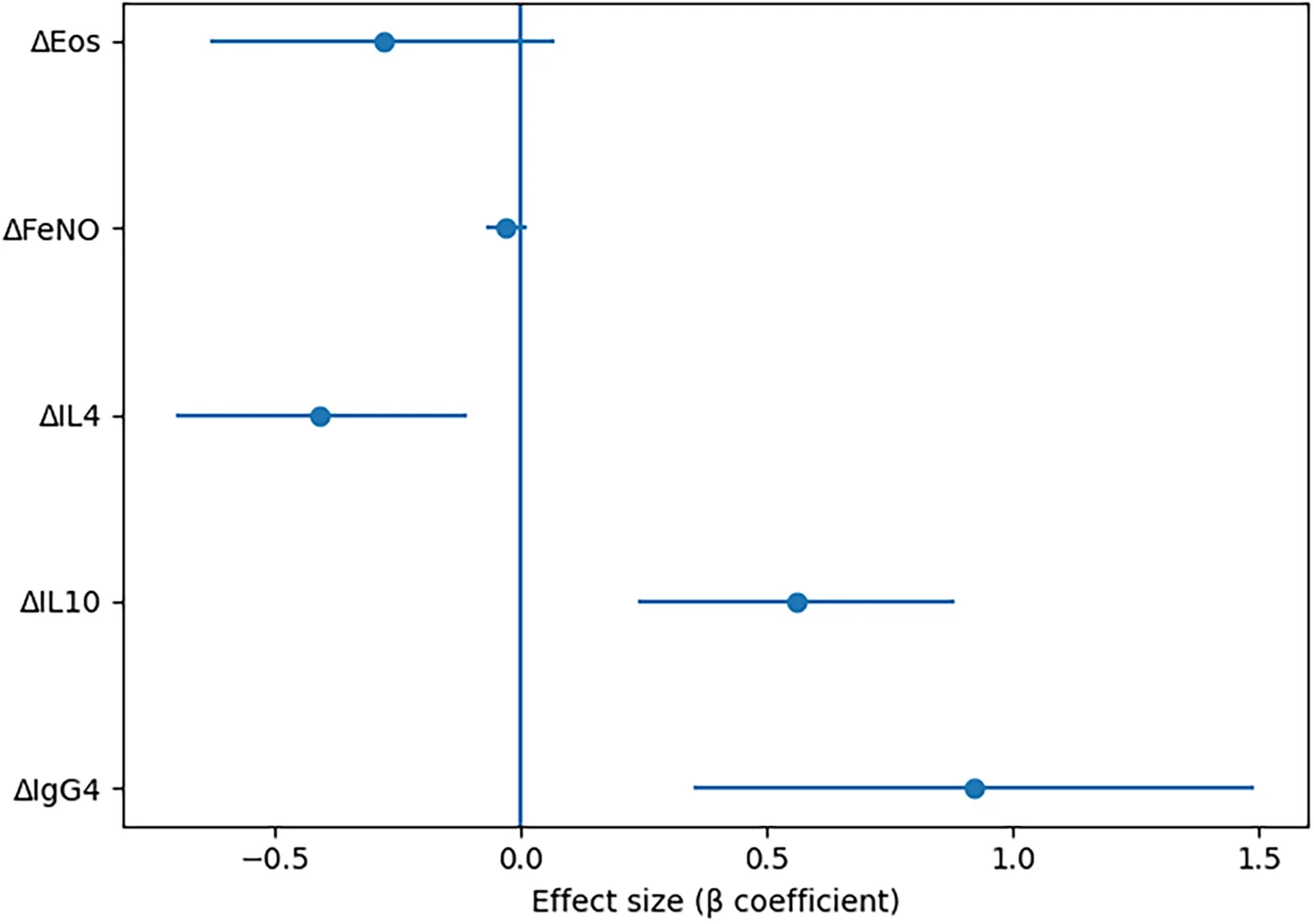
Exploratory associations between clinical and immunological variables and changes in ACT score from baseline to 12 months. The forest plot presents regression coefficients and 95% confidence intervals from the multivariable linear regression model. The vertical reference line indicates no association (*β* = 0). Biomarker variables are expressed as changes from baseline to 12 months. Positive coefficients indicate associations with greater improvement in ACT score.

### Exploratory factors associated with treatment response

An exploratory multivariable logistic regression analysis was performed to examine factors associated with achievement of well-controlled asthma at 12 months, defined as an ACT score of ≥20 (Table 5). In this exploratory model, increases in IgG4 and IL-10 and reductions in IL-4 were associated with achievement of well-controlled asthma. Age, disease duration, FeNO reduction, and treatment group were not significantly associated with the outcome. Given the limited number of treatment-response events relative to the number of candidate variables, these findings should be interpreted as exploratory associations rather than validated predictors. In particular, treatment group was not significantly associated with achievement of well-controlled asthma; this result does not demonstrate equivalent efficacy between SCIT and SLIT.

**Table 5:** Exploratory multivariable logistic regression of factors associated with achievement of well-controlled asthma at 12 months.

Predictor and coding	OR	95% CI	BH-adjusted *P* value
Age, per 1-year increase	1.06	0.92–1.22	0.42
Disease duration, per 1-year increase	0.88	0.70–1.10	0.26
IgG4 increase, yes vs. no	3.21	1.72–5.98	<0.001
IL-10 increase, yes vs. no	2.84	1.54–5.22	0.001
IL-4 decrease, yes vs. no	1.91	1.22–3.00	0.004
FeNO decrease, yes vs. no	1.32	0.92–1.88	0.13
Treatment group, SCIT vs. SLIT	1.12	0.61–2.05	0.71

Treatment response was defined as an ACT score of ≥20 at 12 months, indicating well-controlled asthma. Biomarker changes were categorized according to the direction of change from baseline to 12 months. Odds ratios represent the adjusted association between each listed predictor and achievement of well-controlled asthma. This model was exploratory, included a limited number of treatment-response events relative to the number of candidate variables, and was not intended for individual prediction or clinical decision-making. *P* values were adjusted for multiple comparisons using the Benjamini–Hochberg false-discovery-rate procedure. A two-sided adjusted *P* value <0.05 was considered statistically significant.

### Safety evaluation

The safety profile of allergen immunotherapy was evaluated by analyzing treatment-related adverse events (Table 6). Overall, both immunotherapy modalities were well tolerated. Local reactions occurred in 9 patients (17.0%) in the SCIT group and 8 patients (11.1%) in the SLIT group, with no significant difference between groups (*P* = 0.495). Systemic reactions were rare, occurring in 3 patients in each group, and were mild in severity. No severe systemic reactions or treatment-related hospitalizations were reported during the study period. These findings indicate that both SCIT and SLIT demonstrated favorable safety profiles in children with house dust mite–induced allergic asthma.

**Table 6:** Safety evaluation of immunotherapy.

Event	SCIT (*n* = 53)	SLIT (*n* = 72)	BH-adjusted *P* value
Local reactions	9 (17.0%)	8 (11.1%)	0.495
Systemic reactions	3 (5.7%)	3 (4.2%)	1

*P* values were adjusted for multiple comparisons using the Benjamini–Hochberg false-discovery-rate procedure. A two-sided adjusted *P* value <0.05 was considered statistically significant.

## Discussion

The current study evaluated the clinical efficacy and immunological effects of HDM allergen immunotherapy in children with allergic asthma, with particular attention to the comparison between SCIT and SLIT. After 12 months of treatment, both groups demonstrated significant improvements in asthma control, pulmonary function, and inflammatory biomarkers. Importantly, the magnitude of improvement was comparable between the two treatment modalities. In addition, several immunological indicators—particularly increases in IgG4 and IL-10 together with reductions in IL-4—were closely associated with clinical improvement. These findings describe similar short-term clinical and immunological changes in the two treatment groups during the 12-month observation period. They do not establish the long-term comparative effectiveness or disease-modifying effects of SCIT and SLIT.

The clinical improvements observed in this study are consistent with the established therapeutic benefits of allergen immunotherapy. Compared with conventional pharmacological therapy, which mainly focuses on controlling airway inflammation and bronchoconstriction, allergen immunotherapy aims to modify the underlying allergic immune response and promote long-term immune tolerance to allergens. Over the past decade, increasing evidence has confirmed that AIT can reduce symptom severity, improve asthma control, and decrease airway inflammation in patients with allergic respiratory diseases (19–21). In the present cohort, the mean ACT increase exceeded 4.5 points in both treatment groups, which is greater than the commonly used 3-point minimal clinically important difference and therefore indicates a clinically meaningful improvement in asthma control (22). The 8–9 percentage-point improvements in predicted FEV1 in both groups also indicate improved airflow during the 12-month observation period. However, no universally accepted minimal clinically important difference has been established for FEV1% predicted in children. Therefore, these pulmonary-function changes should be interpreted alongside patient-reported asthma control and other clinical outcomes, rather than as stand-alone measures of clinical benefit. The similar improvements observed in the two groups should not be interpreted as evidence of therapeutic equivalence because of the retrospective, non-randomized design and limited sample size.

Another important observation in this study is the significant reduction in inflammatory markers after immunotherapy. Both FeNO levels and peripheral blood eosinophil counts decreased significantly during treatment, indicating attenuation of eosinophilic airway inflammation. FeNO is widely recognized as a non-invasive biomarker of type-2 airway inflammation and has been shown to correlate with asthma activity and response to therapy. Previous studies have demonstrated that successful allergen immunotherapy is often accompanied by reductions in FeNO and eosinophil levels, reflecting decreased allergic airway inflammation (23, 24). However, FeNO may be influenced by corticosteroid exposure and other clinical factors. Because the retrospective dataset did not contain detailed longitudinal information on inhaled or systemic corticosteroid use, the observed FeNO reductions cannot be attributed solely to immunotherapy and should be interpreted cautiously.

The immunological changes observed in this study are consistent with established concepts of AIT-induced immune modulation. Allergen-specific IgG4 may function as a blocking antibody by competing with IgE for allergen binding, thereby reducing IgE-dependent activation of mast cells and basophils. In the present cohort, IgG4 increased significantly after treatment, showed the strongest positive correlation with *Δ*ACT (*r* = 0.48, *P* < 0.001), and remained positively associated with *Δ*ACT in the multivariable model (*β* = 0.92, *P* = 0.002). These findings are consistent with an association between increased allergen-specific IgG4 and improved asthma control during immunotherapy (25, 26). Cytokine changes provided complementary evidence of altered immune regulation. IL-4 promotes type-2 allergic inflammation and supports IgE class switching, whereas IL-10 is associated with regulatory immune responses and the development of allergen tolerance (9, 26). In this cohort, *Δ*IL-10 was positively correlated with *Δ*ACT (*r* = 0.41, *P* < 0.001) and remained positively associated with *Δ*ACT in the multivariable model (*β* = 0.56, *P* < 0.001). Conversely, *Δ*IL-4 was negatively correlated with *Δ*ACT (*r* = −0.37, *P* < 0.001) and remained negatively associated with *Δ*ACT after multivariable adjustment (*β* = −0.41, *P* = 0.008). Together, these observations are consistent with a shift away from type-2 inflammatory activity and toward a more regulatory immune profile during the 12-month treatment period (27).

A key aspect of this study is the direct comparison between SCIT and SLIT. Both treatment approaches are recommended for allergic respiratory diseases, but their relative effectiveness in children with HDM-induced allergic asthma remains uncertain. Recent systematic reviews and meta-analyses in pediatric populations have reported broadly comparable clinical efficacy of SCIT and SLIT, although direct comparative evidence remains limited and heterogeneous. A recent meta-analysis focused on pediatric asthma found that both modalities were generally associated with clinical benefit, while emphasizing the need for larger, long-term comparative studies (28). Similarly, a systematic review and meta-analysis in children with allergic rhinitis found no significant difference in symptom or medication scores between SCIT and SLIT in direct comparisons, although SLIT was associated with fewer treatment-related adverse events (29). However, differences in study populations, allergens, treatment protocols, outcomes, and follow-up durations limit direct extrapolation of these findings to children with HDM-induced allergic asthma. In the present retrospective cohort, baseline-adjusted analyses showed no statistically significant between-group differences in ACT score, FEV1, FVC, IgG4, IL-10, IL-4, FeNO, or blood eosinophil count at 12 months. These findings indicate that the measured outcomes changed similarly in the two treatment groups over the 12-month observation period. However, the non-randomized design, limited sample size, and absence of an equivalence or non-inferiority framework mean that these findings should not be interpreted as evidence of equivalent efficacy. Longer prospective comparative studies are needed to determine whether any between-group differences are clinically meaningful and sustained over longer treatment periods.

From a clinical perspective, the choice between SCIT and SLIT should be individualized through shared decision-making rather than determined solely by the present findings. For SCIT, families should consider the feasibility of regular clinic visits, repeated injections, and post-injection observation. For SLIT, the feasibility of daily home administration and sustained caregiver-supported adherence should be considered. Treatment selection should also take account of the child's and family's preferences, prior treatment experience, safety considerations, and local availability of each modality. In the present cohort, both modalities were well tolerated, and no significant between-group difference was observed in the recorded local or systemic adverse events. However, because this study was retrospective, non-randomized, and not designed as an equivalence or non-inferiority trial, the similar 12-month changes observed between groups should not be interpreted as evidence that the two modalities are interchangeable. Prospective comparative studies with longer follow-up are needed before definitive treatment-selection recommendations can be made.

The associations between immunological changes and clinical improvement should be interpreted cautiously. Although increases in IgG4 and IL-10 and reductions in IL-4 were associated with greater ACT improvement in the exploratory multivariable model, these findings do not establish causal or mediating mechanisms. The biomarker changes may reflect parallel immunological and clinical responses to immunotherapy, baseline disease characteristics, treatment adherence, changes in concomitant medication, or other unmeasured factors. In addition, measurements were obtained at baseline and 12 months only; therefore, the temporal sequence between biomarker changes and clinical improvement could not be determined. The study measured circulating biomarkers only and did not directly assess regulatory T-cell activity, allergen-specific T-cell responses, or basophil activation. Consequently, these markers cannot yet be used for routine prediction of treatment response or for selection between SCIT and SLIT. Prospective studies with repeated mechanistic assessments are needed to clarify their biological relevance and potential causal role (27).

Several limitations should be considered. First, this was a retrospective, non-randomized cohort study. Treatment modality was determined during routine clinical care rather than allocated by the investigators. Therefore, selection bias and residual confounding from unmeasured treatment-selection factors cannot be excluded. The comparative findings should consequently be interpreted as associations rather than causal estimates of treatment effects. Second, the sample size was limited to the available retrospective cohort. Although both groups improved over 12 months, the study may have been underpowered to detect small but clinically meaningful between-group differences, and a Type II error cannot be excluded. Therefore, non-significant between-group findings should not be interpreted as evidence of therapeutic equivalence. The limited number of treatment-response events relative to the number of candidate variables may have resulted in overfitting of the exploratory logistic regression model. Third, the study could not account for changes in concomitant asthma medications during follow-up, which may have influenced the observed clinical outcomes. Detailed longitudinal information on medication class, dose, frequency, and changes over time was unavailable in the retrospective records. Therefore, a standardized medication-use score could not be calculated, and medication-related confounding could not be adjusted for. Adherence was also assessed retrospectively and was not quantified using a validated instrument. In particular, FeNO analyses were not adjusted for inhaled or systemic corticosteroid exposure or for other potential determinants, such as age and sex. Therefore, residual confounding may have influenced the observed FeNO changes. Fourth, the 12-month follow-up period does not permit assessment of sustained benefit during longer treatment courses, long-term disease modification, or post-treatment effects. Finally, the observed associations between changes in IgG4, IL-10, IL-4, and asthma control do not establish causal or mediating mechanisms. Larger prospective studies with standardized treatment protocols, prespecified sample-size calculations, medication monitoring, validated adherence measures, repeated biomarker assessments, and longer follow-up are needed.

In conclusion, in this retrospective cohort of children with HDM-induced allergic asthma, both SCIT and SLIT were associated with improvements in asthma control, pulmonary-function measures, inflammatory markers, and selected immunological parameters over 12 months. No statistically significant between-group differences were observed in the measured changes during this period. However, the limited sample size, non-randomized retrospective design, and 12-month follow-up preclude conclusions regarding therapeutic equivalence, long-term disease modification, or post-treatment effects. Longer prospective studies are needed to evaluate the durability and comparative long-term efficacy of SCIT and SLIT.

## Data Availability

The raw data supporting the conclusions of this article will be made available by the authors, without undue reservation.
